# The application of gastrointestinal endoscopy in children: a narrative review

**DOI:** 10.3389/fped.2025.1691692

**Published:** 2025-11-24

**Authors:** Guo Zhang, Guiping Zhao, Peng Li, Shutian Zhang

**Affiliations:** Department of Gastroenterology, Beijing Friendship Hospital, Capital Medical University, Beijing, China

**Keywords:** endoscopy in children, gastrointestinal, endoscopic retrograde cholangiopancreatography, endoscopic ultrasound, endoscope dimensions, anesthesia

## Abstract

Digestive endoscopy in children is increasingly used for the diagnosis and treatment of a broad range of diseases affecting the stomach, intestines, biliary tract, and pancreas, with the advantages of being minimally invasive and efficient. Endoscopic procedures in children differ from those in adults in terms of both indications and primary objectives. Furthermore, ensuring the safety and comfort of children during the examination necessitates additional considerations, such as the use of appropriately sized endoscopes, carefully tailored sedation protocols, and bowel preparation regimens. This article provides an overview of the diagnostic value of endoscopy in common digestive tract diseases and challenging conditions in children, and it details the clinical applications of various endoscopic therapeutic techniques. Furthermore, the review focuses on several core aspects of endoscopy in children, including age-stratified selection strategies for endoscopic instruments, safety evaluations of sedation and anesthesia protocols, indications and contraindications for various endoscopic techniques, potential procedure-related adverse events, as well as current disparities in the development of endoscopy in children across different regions. Despite substantial progress in the field, challenges remain, including the lack of specialized devices, technical complexity, and gaps in operator training and quality control. Future efforts should emphasize multicenter studies, the development of standardized operating guidelines, and the integration of artificial intelligence and novel imaging technologies to optimize the endoscopy diagnostic and therapeutic system, thereby advancing digestive endoscopy in children toward greater precision, safety, and efficiency.

## 1 Introduction

Endoscopic technology has become an important tool for diagnosing and treating digestive diseases in children, combining the advantages of being minimally invasive and highly efficient (1). Technically speaking, patient safety and comfort must be considered during the procedure, and therefore certain additional conditions must be met (2). Since the application of endoscopic technology in children began in the 1970s, endoscopic techniques have continued to evolve. In recent years, complex endoscopic procedures such as endoscopic retrograde cholangiopancreatography (ERCP), endoscopic ultrasound (EUS), and peroral endoscopic myotomy (POEM) have been progressively applied to children and has demonstrated favorable safety and efficacy (3–6).

**Figure 1:**
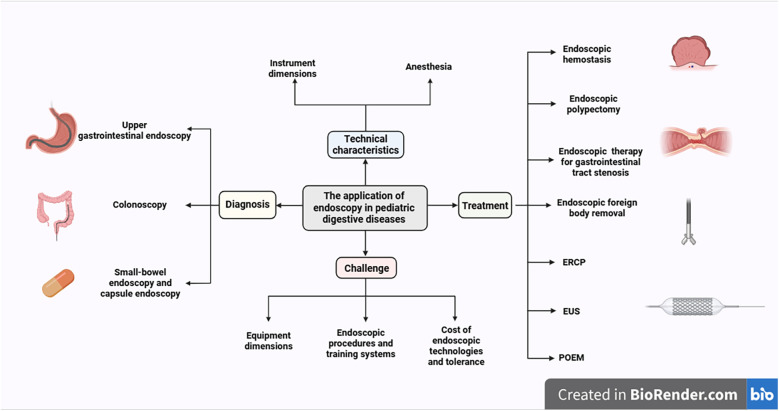
Current applications of gastrointestinal endoscopy in children. (created with BioRender.com).

The field of endoscopy in children continues to face significant challenges, including imperfect implementation of quality standards, a paucity of specialized and standardized assessment tools, and inadequate device compatibility, despite technical advancements that offer minimally invasive options for children (7). This review aims to summarize the clinical applications of existing children's endoscopic techniques, explore future development directions, and promote the precision and individualization of children's endoscopic diagnosis and treatment (Figure 1).

## 2 Methods

### 2.1 Literature search strategy

A systematic literature search was performed using the PubMed database.

### 2.2 Search terms

The search strategy incorporated a combination of Medical Subject Headings (MeSH) and free-text terms to maximize retrieval. Key search concepts and terms include:

Core Topics: endoscopy in children, gastrointestinal endoscopy, children, endoscopic retrograde cholangiopancreatography (ERCP), endoscopic ultrasound (EUS), dimensions.

Diagnosis: diagnosis, diagnostic yield, esophagogastroduodenoscopy (EGD), colonoscopy, small-bowel endoscopy, capsule endoscopy.

Therapeutics: therapeutic endoscopy, foreign body removal, dilation, hemostasis, polypectomy.

Specific Conditions: inflammatory bowel disease (IBD), eosinophilic esophagitis (EoE), polyposis syndrome.

### 2.3 Inclusion criteria

Study Types: Randomized controlled trials, prospective cohort studies, large-scale retrospective studies, systematic reviews, and meta-analyses. Clinical practice guidelines from leading societies (e.g., NASPGHAN, ESPGHAN, ESGE, ASGE) were prioritized.

Population: Studies had to involve a patient population aged ≤18 years.

Publication Date: The search primarily included literature published between January 2008 and August 2025, to cover evidence and technological advances from the past 15 years.

## 3 Technical characteristics of endoscopy in children

The technical execution of endoscopy in children necessitates careful attention to instrument dimensions; in contrast to adults, the selection of endoscopic devices is tailored to the child's somatotype (1). In infants and young children with a body weight below 10 kg, the use of an ultra-thin gastroscope (outer diameter 5–6 mm) is recommended to minimize airway compression and mucosal injury (2); for weight between 10 and 20 kg, an 8 mm small-diameter gastroscope may be selected (8). In ERCP procedures, children weighing more than 10 kg may use standard adult duodenoscopes with an outer diameter of 11.3 mm (9); those weighing less than 10 kg should use specific duodenoscopes for children with an outer diameter of 7.5 mm (10). EUS is generally indicated only for children exceeding 15 kg; smaller children may be considered to substitute with a bronchial ultrasound probe (10). In addition, endoscopic therapy accessories (e.g., dilating balloons and hemostatic clips) should be matched to the luminal dimensions of children to prevent iatrogenic injury (3).

Endoscopy anesthesia is a critical component to ensure smooth procedures and patient safety. In recent years, multiple sedation regimens and techniques have been applied in clinical practice to optimize sedation efficacy and reduce adverse events. Studies indicate that endoscopy in children often requires deep sedation or general anesthesia, especially for younger or high-risk children and for advanced endoscopic procedures (10, 11). Common sedatives include propofol, midazolam, ketamine, and dexmedetomidine; among these, propofol is widely used due to its rapid onset and quick recovery, but its potential to cause respiratory depression should be noted (12). Dexmedetomidine, when delivered via inhalation, can significantly suppress gag and cough reflexes, reduce intraoperative anesthetic requirements, and improve endoscopist satisfaction with favorable safety (13). However, Johnson et al. found that low-dose dexmedetomidine combined with propofol did not reduce total propofol consumption and was associated with increased postoperative hypotension and prolonged recovery (14). Shafa et al. reported that ketamine combined with lidocaine did not significantly improve hemodynamics but could reduce the dose requirements of a single agent (12). Emerging agents such as remimazolam have shown rapid metabolism and good safety in adults, but pediatric data are still limited (15). Overall, sedation management for endoscopy in children should be individualized, balancing age, weight, and comorbidities to select the best plan, with emphasis on teamwork and postoperative follow-up (1).

## 4 Gastrointestinal endoscopy in the diagnosis of children

### 4.1 Esophagogastroduodenoscopy (EGD)

For children, EGD serves as a primary tool for investigating organic diseases. It is crucial for identifying the underlying causes of non-specific symptoms—such as failure to thrive, chronic abdominal pain, recurrent vomiting, and unexplained anemia—and for diagnosing specific conditions, notably gastroesophageal reflux disease (GERD) and eosinophilic esophagitis (EoE) (16, 17). Endoscopic examination allows direct visualization of esophageal mucosal lesions, including erosions, ulcers, or strictures, and can be combined with biopsies for histologic assessment, thereby enhancing diagnostic accuracy (18). For children with GERD, endoscopy can reveal varying degrees of esophagitis, and the strong correlation between pH monitoring and endoscopic findings (Boix-Ochoa score) further validates the diagnostic value of endoscopy (18). Additionally, endoscopy is particularly critical in differentiating EoE from GERD; the diagnosis of EoE can be confirmed by identifying eosinophilic infiltration in the esophageal mucosa (≥15 eosinophils per high-power field) on biopsy (19). In refractory cases, endoscopy can guide therapeutic decisions; for example, Rizvi et al. observed that IgG positivity in esophageal biopsy specimens may aid in detecting response to proton pump inhibitor (PPI) therapy or monitoring disease progression in children with EoE (20).

EGD in children differs from adult practice in several key aspects. A primary distinction lies in biopsy practices: children routinely undergo multi-site biopsies, even in the absence of macroscopic mucosal abnormalities, to avoid missing clinically subtle mucosal diseases. In contrast, biopsies in adults are typically targeted and obtained only from suspicious lesions (9, 10). Secondly, there are divergent focuses in diagnostic indications. In children, EGD is primarily employed to diagnose conditions such as failure to thrive, eosinophilic esophagitis, and inflammatory bowel disease. In adults, the procedure is more often oriented toward screening for malignancies and evaluating conditions like esophageal varices (9, 10).

Transnasal endoscopy (TNE) represents a novel, sedative-free modality for upper GI assessment, particularly advantageous for longitudinal surveillance in children with EoE, GERD, and postoperative esophageal procedures (21, 22). In cohorts of children, TNE demonstrates satisfactory tolerability, shortened procedure times, and biopsy adequacy comparable to conventional endoscopy, enabling accurate quantification of esophageal inflammatory activity (23, 24). Additional strategies, such as video goggle or VR-based unsedated TNE, may further reduce anxiety and improve diagnostic yield (25–27).

### 4.2 Colonoscopy

Studies have shown that the positive diagnostic yield of colonoscopy in children can exceed 70%, with inflammatory bowel disease (including ulcerative colitis and Crohn's disease) representing the most common diagnosis, accounting for over 40% (28). In addition, colonoscopy can effectively identify conditions such as polyposis and vascular malformations in children, providing important guidance for clinical management (29). For children who have undergone solid organ transplantation (SOT) or hematopoietic stem cell transplantation (HSCT), colonoscopy can reliably detect post-transplant lymphoproliferative disorders (PTLD), infectious colitis, and graft-vs. -host disease (GVHD) (30, 31).

Colon capsule endoscopy (CCE) is a wireless, ingestible mini-endoscope used for the non-invasive examination of the colon. In conditions involving children such as ulcerative colitis, CCE demonstrates high accuracy for assessing disease activity and extent, with reported sensitivity of 95% and specificity of 100%, outperforming modalities like intestinal ultrasound and fecal calprotectin (32, 33). Its advantages include the avoidance of anesthesia, excellent patient tolerance, and its utility as a complementary tool to colonoscopy, particularly for long-term monitoring in children, which can reduce the frequency and risks associated with invasive procedures. However, limitations exist, such as the inability to obtain biopsies or perform therapeutic interventions, a high dependency on adequate bowel preparation, the potential for capsule retention, and substantial cost (32, 33).

In terms of technical performance, colonoscopy in children demonstrates a high success rate, typically exceeding 90%, with relatively low complication rates (34). While adult colonoscopy is primarily utilized for colorectal cancer screening and surveillance, colonoscopy in children is chiefly indicated for diagnosing conditions such as inflammatory bowel disease (IBD) and polyposis syndromes. Consequently, a critical procedural step in children is intubation of the ileocecal valve, which allows for endoscopic examination and biopsy of the terminal ileum (9, 10, 28). This maneuver is critical for the differential diagnosis of IBD, as pathologies like Crohn's disease frequently involve the terminal ileum, making examination of the colon alone insufficient for a comprehensive assessment. Successful ileal intubation thereby enables a definitive diagnosis and accurate disease mapping. Studies confirm that high rates of both cecal and ileal intubation (exceeding 90% for each) are achievable in colonoscopy for children (35), Ileocecal intubation rate should be regarded as a key quality indicator for colonoscopy in children. To enhance safety and diagnostic accuracy, the choice of colonoscope should be tailored to the child's age and weight (8, 28). Through colonoscopy, clinicians can directly visualize mucosal abnormalities and, when combined with histopathology, establish a definitive diagnosis to guide individualized treatment (9, 29).

Adequate bowel preparation is a critical step to ensure the smooth performance of colonoscopy (36). Current commonly used bowel cleansing agents include polyethylene glycol (PEG) and sodium picosulfate magnesium citrate (SPMC); there is no significant difference in cleansing efficacy between them (37, 38). PEG requires high-dose administration and may be poorly tolerated, whereas SPMC has a smaller volume and better palatability, making it more acceptable to children (39).Moreover, the use of SPMC can reduce the need for nasogastric tube insertion, thereby alleviating discomfort for children (37). Split-dose regimens are associated with markedly improved bowel cleanliness compared with day-before dosing, and patient education along with adjunctive tools (e.g., smartphone applications) can further enhance bowel preparation quality (37, 39).

### 4.3 Small-bowel endoscopy and capsule endoscopy

Small-bowel endoscopy, including push enteroscopy (PE) and device-assisted enteroscopy (primarily balloon-assisted enteroscopy, BAE) are pivotal minimally invasive techniques for small bowel disorders in children (40). PE employs a push-and-pull method with 150–250 cm working-length endoscopes, targeting proximal small bowel lesions such as polyps and Crohn's disease (CD) in children ≥2 years and ≥10 kg (40). BAE enhances access via balloon-anchored pleating: single-balloon enteroscopy (SBE) uses a single overtube balloon (≥3 years, ≥13.5 kg), while double-balloon enteroscopy (DBE) employs dual balloons (≥2 years, ≥12 kg), reaching mid-to-distal segments (40). Both techniques address core indications: obscure gastrointestinal bleeding (OGIB), CD, and polyposis syndromes (e.g., Peutz-Jeghers syndrome, PJS), with BAE enabling therapeutic interventions like polypectomy and stricture dilation (41, 42). PE offers cost-effectiveness and wide availability, while BAE provides superior diagnostic yields (58.8%–78.6% for DBE) and therapeutic versatility, reducing surgical reliance (40, 41). Safety profiles are favorable, with minor complications (abdominal discomfort) predominating, though younger children (<10 years) have slightly higher risks (40, 41).

Capsule endoscopy (CE) play an important role in children for diagnosis, with particularly high sensitivity for the diagnosis and assessment of CD (33, 41), and enable detection of very early-onset inflammatory bowel disease (VEO-IBD). Studies show that 42% of VEO-IBD patients have small-bowel abnormalities detected by CE, with aphthous ulcers being predominant (43). Moreover, the newly proposed Crohn's disease activity index for CE (CE-CD) demonstrates good reliability and predictive value in children, effectively assessing inflammation and predicting clinical outcomes such as hospitalization and relapse (44). For OGIB, CE can identify etiologies including vascular malformations, ulcers, polyps, and Meckel's diverticulum (29, 45, 46). CE also show high accuracy in screening and monitoring polyposis syndromes such as PJS (41, 42, 47). Some reports indicate that CE can clearly visualize characteristic white villi changes in the small intestine, enabling precise diagnosis of intestinal lymphangiectasia (48, 49). Pan-enteric capsule endoscopy (PCE) is a non-invasive endoscopic technique that allows for the simultaneous examination of both the small bowel and colon. It holds significant value in Crohn's disease in children by providing a complete assessment of mucosal inflammation, thereby guiding treat-to-target strategies and improving rates of mucosal healing (50). Its key advantage over traditional capsule endoscopy lies in its ability to provide a comprehensive, one-stop evaluation of the entire bowel, thereby eliminating the need for repeated procedures (50).

CE use in children carries a risk of capsule retention, with reported rates ranging from 0.18% to 2%, and the risk may be higher in CD patients due to intestinal strictures (51–53). Clinically, when intestinal stricture is suspected, a patency capsule (the Agile Patency System) is recommended; this dissolvable capsule of the same size as an endoscopic capsule, equipped with an internal localization marker, can precisely identify the location of strictures and prevent retention (54). To reduce blind spots and missed lesions during CE, magnetically controlled capsule endoscopy (MCE) can achieve precise repositioning via external magnetic guidance, thereby improving safety and sensitivity (55–58). Difficulty swallowing the CE is not uncommon in children due to age, developmental stage, or psychological factors. To address this challenge, endoscopic-assisted delivery has become a well-established solution, primarily utilizing a dedicated delivery device, a transparent cap, or a retrieval basket (59). Evidence confirms that this technique ensures precise capsule placement in the descending duodenum, effectively prevents gastric retention, and significantly increases the completion rate of full small-bowel examination to over 94% (53). Regarding safety, multiple large-scale studies have reported a capsule retention rate of less than 3% with no serious adverse events, demonstrating a favorable risk-benefit profile, particularly for young or swallowing-impaired children (55, 60).

## 5 Gastrointestinal endoscopy in the treatment of children

### 5.1 Endoscopic hemostasis

Endoscopic therapy plays a key role in the management of gastrointestinal bleeding in children, with the goals of hemostasis and prevention of rebleeding. For variceal hemorrhage, endoscopic variceal ligation (EVL) is the treatment of choice, providing effective hemostasis with relatively low complication rates (61, 62). In children with esophageal varices, endoscopic sclerotherapy may be used to eradicate varices and achieve hemostasis (63). For nonvariceal bleeding, endoscopic injection of epinephrine combined with mechanical or thermal coagulation (e.g., argon plasma coagulation or metallic clips) can achieve hemostasis (61). Titanium clips are currently the preferred mechanical modality for nonvariceal gastrointestinal bleeding (61). In the very rare Dieulafoy lesions in children, endoscopic therapy is first-line and may include clipping, thermal coagulation, sclerosants, epinephrine injection, or laser therapy (64–66). The advantages of endoscopic treatment include rapid localization of the bleeding source and precise intervention, which significantly reduces the need for surgery and length of hospitalization. Treatment should be individualized according to the child's age and clinical status (61).

Compared with adult endoscopic treatment for gastrointestinal bleeding, etiologically, children predominantly present with juvenile polyps, allergic colitis, or vascular anomalies, whereas adults more commonly have diverticulitis or colorectal cancer (29, 61). Children's cases require a greater reliance on deep sedation or general anesthesia due to children's limited cooperation (67). In addition, we must consider the following special safety aspects for children: meticulous hemodynamic monitoring given children's more fragile physiology, tailored bowel preparation to avoid adverse effects, and family-centered communication to support informed consent (61, 67). Furthermore, endoscopists should prioritize minimizing tissue trauma and ensuring procedural efficiency to reduce complications, reflecting the distinct clinical needs of the population of children (67, 68).

### 5.2 Endoscopic polypectomy

Endoscopy is also employed for polypectomy, especially for symptomatic polyps and polyposis syndromes (69). There is currently no unified guideline for polypectomy techniques in children. For small polyps (≤5 mm), cold forceps polypectomy (CFP) or cold snare polypectomy (CSP) are commonly employed, with CSP preferred due to higher complete resection rates and lower complication risk (70, 71). For largerpolyps or pedunculated polyps, endoscopic mucosal resection (EMR) or endoscopic submucosal dissection (ESD) can improve safety, though the choice should be tailored to the individual child (72, 73). In children with PJS, BAE enables safe and effective small-bowel polypectomy, with high symptom relief, reduced risk of intussusception and surgical intervention (74, 75). Some studies suggest that endoscopic ischemic polypectomy (EIP) is associated with fewer complications than conventional polypectomy, making it particularly suitable for children with PJS (76).

Children and adults differ substantially in polyp characteristics, indications, and endoscopic polypectomy techniques. Polyps in children are mostly benign pedunculated juvenile polyps (highly vascularized), while adults have more sessile adenomas with malignant potential (69). Procedures for children are symptom-driven (e.g., rectal bleeding), unlike adult screening-focused practices (69). Technically, children often use cold/hot snare polypectomy, with hot snare preferred for larger polyps, whereas adults prioritize cold snare for <10 mm lesions (69, 72). Critical safety concerns of children include thinner bowel walls, increased bleeding risk from vascular polyps, limited exposure to advanced techniques, and the need to minimize tissue injury—requiring tailored approaches to avoid thermal damage and ensure complete resection without compromising safety (69, 76).

### 5.3 Endoscopic therapy for gastrointestinal tract stenosis

Endoscopic therapy can also be used for children with gastrointestinal strictures, especially esophageal anastomotic stricture and EoE-related stricture (77). For esophageal stricture, endoscopic balloon dilation (EBD) is the first-line treatment, with a low complication rate (e.g., perforation) but requiring multiple sessions to maintain long-term esophageal patency (78). For refractory stenosis, adjunctive local steroid injections (intralesional steroid injection, ISI) can reduce inflammation and scar formation (79). Additionally, stent implantation provides sustained dilation and is suitable for long-segment strictures or recurrent cases, though risks such as stent migration and mucosal injury should be noted (80, 81). For severe fibrotic strictures, endoscopic electrocautery incisional therapy (EIT) can incise scar tissue to improve dilation outcomes (82). For complex strictures, endoscopic magnetic compression anastomosis (MCA), a minimally invasive option, has been demonstrated as effective, offering a minimally invasive solution for complete occlusion or extreme narrowing of the esophagus by endoscopically guiding placement of magnets to progressively reconstruct the lumen (83). Overall, endoscopy can improve prognosis through multiple therapeutic approaches and reduce the need for open surgery.

Compared with adult endoscopic management of gastrointestinal strictures—often associated with neoplasia or chronic conditions—practice in children emphasizes etiologies such as EoE or congenital anomalies (77). Children-specific safety considerations include: heightened anesthesia-related risks, with potential impacts on neurodevelopment from repeated exposure; reliance on combined barium esophagrams to minimize missed strictures; and trauma prevention in smaller luminal diameters—unlike adults, where perforation is the principal concern (77, 84). Additionally, Strictures in children frequently necessitate more frequent dilations, requiring closer long-term monitoring of outcomes.

### 5.4 Endoscopic foreign body removal

Endoscopy demonstrates high efficiency and safety in gastrointestinal foreign body retrieval for children, with a low complication rate (85). Endoscopy enables precise localization of the foreign body and, depending on its characteristics, safe extraction using various accessories (e.g., graspers, retrieval nets). This is especially advantageous for high-risk objects such as sharp foreign bodies or batteries, where it can help prevent mucosal injury or perforation (86, 87). According to Oliva and colleagues, the urgency of endoscopy is categorized into four levels: emergency (<4 h), urgent (<24 h), early elective (<48 h), and elective (>48 h), depending on the type and location of the foreign body and the child's clinical symptoms (88). For example, button batteries in the esophagus require emergent removal within 2 h to avoid serious complications (88, 89). Although endoscopic techniques are well established, children with repeated or deliberate ingestion of foreign bodies often have underlying psychiatric disorders, necessitating multidisciplinary collaboration, including psychological interventions (90).

### 5.5 Application of ERCP in children

Although many noninvasive diagnostic modalities have supplanted ERCP for diagnosis in children, ERCP demonstrates relatively high safety and efficacy in the treatment of biliary and pancreatic disorders in children (91). Indications for ERCP in children mainly include choledocholithiasis, congenital biliary dilatation (CBD), postoperative complications (e.g., biliary leak, pancreaticopleural fistula), and recurrent pancreatitis, among others (92–94). Several meta-analyses indicate that the overall treatment success rate of ERCP in children ranges from approximately 74% to 95%, with stent placement being the most common therapeutic modality; other approaches include sphincterotomy, stone extraction, dilatation procedures (sphincterotomy and balloon dilation), and balloon dilatation, etc.

The postoperative adverse event rate is about 7%–8%, with postoperative pancreatitis being the most frequent complication (95, 96). Other rare complications include biliary infection, and bleeding (97, 98). Although intestinal complications are not the most common, they are critically important in ERCP for children, these primarily include intestinal strictures, adhesions, and postsurgical anatomical alterations (98, 99). Such conditions-particularly stenosis or adhesions-can prevent the passage of a standard duodenoscope to the duodenal papilla, increasing the risk of cannulation failure and intestinal perforation. To address these challenges, a comprehensive clinical approach is essential. Preoperative imaging should be used to delineate anatomical details. During the procedure, the selection of specialized equipment—such as smaller-caliber duodenoscopes—should be tailored to the child's weight and the degree of stenosis. In cases of severe stricture, balloon dilation may be considered. Crucially, these procedures should be performed in specialized centers equipped with anesthesiology support for children, onsite surgical backup, and endoscopists experienced in managing complex cases in children to maximize safety and manage potential complications effectively (10). The following summarizes recent results on ERCP in children (Table 1). It should be noted that ERCP in children require general anesthesia (10).

**Table 1:** ERCP in children with digestive disease.

Citation	Publication year	Study period	Number of patients/procedures	Median age (range)	Etiology	Number of therapeutic ERCP-procedures % (*n*)	Success rates % (*n*)	Adverse event % (*n*)
Keane et al. (101)	2018	1992–2014	66/87	14 years (3–17 years)	Chronic or recurrent pancreatitis, pancreatic fluid collections, biliary obstruction, bile leak	100% (87/87)	100% (87/87)	0% (0/87)
Sun et al. (102)	2018	2014–2017	17/17	56.4 months (10–120 months	Congenital biliary dilatation	100% (17/17)	100% (17/17)	5.9% (1/17)
Keil et al. (103)	2019	1999–2018	626/856	4 years and 11 months (12 days-17 years and 6 months)	Biliary obstruction, chronic pancreatitis, pancreaticduct disruption, bile leak	58.8% (503/856)	96% (822/856)	9.35% (80/856)
Asenov et al. (92)	2019	1994–2014	24/24	15 years (6–17 years)	Choledocholithiasis, postoperative complications, recurrent pancreatitis	71% (17/24)	74%	4% (1/24)
Zeng et al. (104)	2019	2008–2019	75/112	6 years (9 months-16 years)	Symptomatic pancreaticobiliary maljunction	100% (112/112)	100% (75/75)	16% (12/75)
Wen et al. (105)	2019	2008–2017	38/74	10 years (2–18 years)	Pancreas divisum	100% (74/74)	93.2% (69/74)	14.9% (11/74)
Shah et al. (106)	2020	2008–2018	110/232	13.3 years	Common bile/pancreatic duct obstruction, pancreatic duct/ common bile duct trauma/leak, pancreas divisum	100% (232/232)	95% (222/232)	6.1 (14/232)
Mercier et al. (97)	2021	2008–2019	271/740	10.9 years (5 days-17 years)	Choledocholithiasis, chronic pancreatitis	90% (423/470)	93.4% (439/470)	24.4% (83/340)
Åvitslandet al. (107)	2021	1999–2017	158/244	8.8 years (8 days-17.9 years)	Biliary atresia, biliary stricture	51.2% (125/244)	92.2% (225/244)	10.8% (24/222)
Goetz et al. (108)	2021	NA	126/135	≤1 years	Biliary atresia	14.3% (18/126)	100% (126/126)	0% (0/126)
Deng et al. (109)	2021	2018–2019	66/92	7.1 years (8 months-14 years)	Chronic pancreatitis, pancreaticobiliary maljunction, pancreas divisum, pancreatic pseudocyst	100% (92/92)	100% (92/92)	20.7% (19/92)
Perera et al. (110)	2022	2015–2020	62/98	11.01 years (3–16 years)	Chronic pancreatitis, biliary diseases	100% (98/98)	85.7% (84/98)	9% (9/98)
Saraiva et al. (111)	2023	1994–2022	57/65	13 years (1–17 years)	Biliary obstruction, lithiasic acute pancreatitis, recurrent pancreatitis	80% (52/65)	95.4% (62/65)	3.1%(2/65)
Gong et al. (112)	2023	1983–2022	31/15	11.71 years (1–18 years)	Traumatic pancreatic injury	60% (9/15)	NA	66.7% (10/15)
Li et al. (113)	2024	2013–2023	76/113	13 years (3 years and 5 months-17 years and 9 months)	Biliary obstruction, chronic pancreatitis	87.6% (99/113)	100% (113/113)	14.2% (16/113)
Çirkin et al. (114)	2024	2017–2021	50/65	12.7 years (1–18 years)	Choledocholithiasis, chronic pancreatitis	NA	92.3% (60/65)	6.1% (4/65)
Wang et al. (115)	2025	2019–2024	58/58	5.7 years (1.4–16.4 years)	Common bile duct dilatation and stones	100% (58/58)	100% (58/58)	19% (11/58)
Poddar et al. (116)	2025	2010–2024	222/286	9.4 years (3 months -17 years)	Choledochal cyst, choledocholithiasis, bile leak, chronic pancreatitis with pancreatic duct stricture	95% (273/286)	92% (204/222)	16% (36/222)
Batıbay et al. (117)	2025	2013–2024	83/153	12.9 years (3–17 years)	Common bile duct stones, biliary hydatid cyst-related complications	100% (153/153)	98.8% (82/83)	18% (15/83)

ERCP, endoscopic retrograde cholangiopancreatography; NA, not available.

Because children are more susceptible to malignant tumors after exposure to ionizing radiation, particular attention must be paid to the risks of radiation exposure. During the procedure, radiation dose should be monitored and radioprotective shielding should be appropriately used to minimize the child's radiation exposure (100).

### 5.6 Application of EUS in children

Endoscopic ultrasound (EUS) can clearly delineate pancreaticobiliary structures, with diagnostic performance superior to conventional imaging for chronic pancreatitis, microlithiasis in the biliary tract, and pancreatic pseudocysts, among other conditions (118). Additionally, EUS can be used to guide fine-needle aspiration biopsy, providing histopathological diagnostic confirmation for pancreatic masses and autoimmune pancreatitis (119). In terms of treatment, EUS-guided drainage can be safely performed for children with symptomatic pancreatic pseudocysts, thereby avoiding surgical intervention (120). For children with biliary obstruction, EUS-guided biliary drainage represents an effective alternative, particularly after ERCP failure (119). In children weighing more than 25 kg, adult-endoscopic ultrasound can be safely utilized (121). In smaller children, ultrasound endoscopic probes through standard children endoscope channels may be employed (9, 122). For children weighing more than 15 kg, EUS can be performed safely under intravenous sedation (e.g., propofol) without general anesthesia (119). Infants and young children, due to smaller anatomical structures, may pose greater technical and anesthesia-related challenges (123). The following summarizes recent studies on EUS in children (Table 2). Overall, EUS demonstrates a high technical success rate (118). The complication rate is low, with most adverse events being mild pancreatitis, bleeding, and infection (100, 118).

**Table 2:** EUS in children with digestive diseases.

Citation	Publication year	Study period	Number of patients	Median age (range)	Etiology	Operation	Success rates % (*n*)	Adverse event % (*n*)
Olmos et al. (124)	2019	2019	1	12 years	Liver cirrhosis and gastric variceal hemorrhage	EUS-guided coil placement and cyanoacrylate embolization	100% (1/1)	0% (0/1)
Ávila et al. (125)	2019	2009–2016	54	16 years (9–17 years)	Recurrent acute pancreatitis, microlithiasis, pancreatic tumors	92.6% diagnostic operation 7.4% therapeutic operation (pancreatic pseudocyst drainages, endoscopic necrosectomy)	Diagnostic EUS: 85% (46/54) Therapeutic EUS: 100% (4/4)	0% (0/54)
Altonbary et al. (126)	2020	2016–2020	13	15.6 years (6–18 years)	Pancreatobiliary disorders, mediastinal lesions, perigastric lesions	46.2% diagnostic operation 53.8% EUS-FNA	100% (13/13)	0% (0/13)
Walsh et al. (127)	2020	NA	2	2 years and 4 years	Pancreatic fluid collections	EUS guided transmural drainage (EUS-TD)	100% (2/2)	50% (1/2)
Ruan et al. (128)	2021	2021	1	16 years	Primary gastric Burkitt's lymphoma	EUS guided fine-needle biopsy (EUS-FNB)	100% (1/1)	0% (0/1)
Piester et al. (129)	2021	2017–2020	98	10.7 years (3–18 years)	Choledocholithiasis, pancreatic fluid collections, chronic and acute recurrent pancreatitis, pancreatic mass, luminal lesions/strictures	75.5% diagnostic operation 15.3% EUS-FNA/FNB 9.2% therapeutic operation	100% (98/98)	1% (1/98)
Barakat et al. (130)	2021	2008–2018	12	15 years (11–18 years)	Gastric variceal bleeding	EUS-guided coil placement	100% (12/12)	0% (0/12)
Ishii et al. (131)	2022	2022	1	7 years	Cholangitis (choledochojejunal anastomotic stricture)	EUS- guided hepaticogastrostomy	100% (1/1)	0% (0/1)
Barakat et al. (132)	2022	2009–2020	279 (306 procedures)	15.7 years (2–18 years)	Pancreaticobiliary region, subepithelial or regional lesion, celiac plexus block, hemostasis	57.8% diagnostic operation 17% EUS-FNA/FNB 25.2% therapeutic operation	Diagnostic EUS: 96.2% (49/52) Therapeutic EUS: 98.7% (76/77)	0% (0/306)
Ragab et al. (133)	2022	2017–2020	29	9 years (2.5–15 years)	Solid pancreatic mass, pancreatic cyst, suspected chronic pancreatitis, pancreatic pseudocyst	44.8% diagnostic operation 37.9% EUS-FNA/FNB 17.3% therapeutic operation	Diagnostic EUS: 87.5% (21/24) Therapeutic EUS: 100% (5/5)	6.9% (2/29)
Dalal et al. (134)	2022	2018–2020	85（92 procedures)	12.1 years (5–18 years)	Choledocholithiasis, cholelithiasis, pancreatic pseudocyst	74.1% diagnostic operation 20% EUS-FNB 5.9% therapeutic operation (EUS-guided rendezvous, EUS-guided cystogastrostomy)	100% (85/85)	0% (85/85)
Yabe et al. (135)	2023	2006–2021	6	12.5 months (2–45 months)	Congenital esophageal or duodenal stenosis	100% diagnostic operation 100% therapeutic operation	100% (6/6)	0% (0/6)
Schwartz et al. (136)	2025	2020–2023	83	16 years (6.9–21 years)	Metabolic dysfunction-associated steatotic liver disease, autoimmune hepatitis and/or primary sclerosing cholangitis, elevated liver enzymes	EUS- guided liver biopsy	100% (83/83)	5% (4/83)

EUS, endoscopic ultrasound; EUS-FNA, EUS-guided fine-needle aspiration; EUS-FNB, EUS guided fine-needle biopsy; NA, not available.

### 5.7 POEM for the treatment of achalasia

POEM is principally used to treat children with achalasia. A meta-analysis shows technical success and clinical success rates of 97.1% and 88–94.4%, respectively, in children, with postoperative Eckardt scores significantly reduced (137). The following summarize recent results on POEM in children with achalasia (Table 3). The procedure is applicable to all types of achalasia, and remains effective even in previously treated or complex cases (e.g., sigmoid-type achalasia) (138, 139). The advantages of POEM include its minimally invasive nature, rapid postoperative recovery, and shorter hospital stay (140, 141). However, the incidence of postoperative gastroesophageal reflux (GER) is relatively high, with about 26.3% of children developing erosive esophagitis, necessitating long-term follow-up and proton pump inhibitor therapy (137). Overall, POEM provides a safe and effective treatment option for children with achalasia, but its technical complexity and postoperative reflux issues warrant careful consideration in clinical practice.

**Table 3:** POEM in children with achalasia.

Citation	publication year	Study period	Number of patients	Median age (range)	Type of achalasia (I, II, III)	Success rates % (*n*)	Postoperative reflux % (*n*)	Adverse event % (*n*)
Nabi et al. (142)	2016	NA	15 (10 completed 1-year follow-up)	14 years (9–18 years)	4,10,1	100% (10/10)	20% (2/10) GERD (gastroesophageal reflux disease)	6.7% (1/15) Mucosal injury 6.7% (1/15) capnoperitoneum 13.3% (2/15) subcutaneous emphysema 20% (3/15) retroperitoneal air
Mejía et al. (143)	2019	2018	1	11 years	0,1,0	100% (1/1)	0% (0/1)	0% (0/1)
Chone ´ et al. (144)	2019	2012–2018	117	14.2 years	36,66,8	90.6% (106/117)	14.5% (17/117) symptomatic GERD 21% (25/117) esophagitis	3.4% (4/117) mucosotomies 1.7% (2/117) subcutaneous emphysema 0.9% (1/117) esopleural fistula
Wood et al. (145)	2020	2014–2019	21	13 years (2–17 years)	11,10,0	100% (21/21)	0% (0/21)	4.8% (1/21) Capnoperitoneum 4.8% (1/21) mucosotomy 9.5% (2/21) subcutaneous emphysema
Saez et al. (146)	2021	2017–2019	5	11.2 years	0,5,0	100% (5/5)	2% (1/5) erosive esophagitis	0% (0/5)
Nabi et al. (147)	2022	2013–2021	69 (38 ≥ 4 years follow-up))	14.7 years (4–19 years)	11,23,1	94.7% (36/38)	13.8% (4/29) symptomatic GERD 57.1% (8/14) erosive esophagitis	NA
Bi et al. (148)	2023	2012–2020	48 (34 with long-term follow-up)	16 years (7–18 years)	7,32,3	97% (35/36)	17.6% (6/34) symptomatic GERD 5.9% (2/34) erosive esophagitis	6.2% (3/48) Mucosal injury 4.2% (2/48) pneumoperitoneum 4.2% (2/48) subcutaneous emphysema

POEM, peroral endoscopic myotomy; GERD, gastroesophageal reflux disease; NA, not available.

## 6 Indications, contraindications, and adverse events of endoscopy in children

Gastrointestinal endoscopy has become an indispensable tool for diagnosing and treating digestive tract and pancreatobiliary diseases in children. Its application emphasizes the principles of being child-centered, safety-first, and purpose-driven. Gastroscopy and colonoscopy serve as the primary modalities for evaluating mucosal lesions in the upper and lower gastrointestinal tract, providing both diagnostic (e.g., for IBD and EoE) and therapeutic (e.g., polypectomy, hemostasis) functions. For the small intestine, which is beyond the reach of traditional endoscopy, capsule endoscopy offers a non-invasive screening method capable of effectively detecting abnormalities. On this basis, device-assisted enteroscopy allows for further examination and treatment of suspicious lesions. In the field of pancreatobiliary diseases, the role of ERCP has shifted entirely from diagnosis to advanced therapeutic interventions such as stone extraction, stent placement, and drainage. Meanwhile, EUS, with its superior imaging and guided puncture capabilities, plays a crucial role in tumor evaluation, cyst drainage, and other areas. Below is a summary of the diagnostic and therapeutic indications for various types of endoscopy (Table 4) (10, 101, 149).

**Table 4:** Indications for various endoscopic techniques in the diagnosis and treatment of digestive diseases in children.

Endoscopic technique	Primary diagnostic indications	Primary therapeutic indications
EGD	Unexplained anemia, weight loss, failure to thrive Recurrent vomiting, dysphagia, odynophagia, chest pain Upper GI bleeding (hematemesis, melena) Suspicion of specific diseases (EoE, celiac disease, H. pylori, IBD) Evaluation following corrosive ingestion	Foreign body retrieval Hemostasis (e.g., ulcers, Dieulafoy's lesion) Band ligation or sclerotherapy of esophageal varices Dilation of esophageal/upper GI strictures Percutaneous endoscopic gastrostomy (PEG) placement
Colonoscopy	Lower GI bleeding (hematochezia) Unexplained chronic diarrhea, iron-deficiency anemia Diagnosis and assessment of Inflammatory Bowel Disease (IBD) Surveillance of polyposis syndromes Evaluation for graft-versus-host disease (GVHD)	Polypectomy Stricture dilation Hemostasis Foreign body retrieval
Small-bowel endoscopy	Obscure Gastrointestinal Bleeding (OGIB) Suspected small-bowel Crohn's disease (when conventional endoscopy/imaging is inconclusive) Surveillance in polyposis syndromes (e.g., Peutz-Jeghers) Suspected small-bowel tumors or ulcers	Polypectomy Hemostasis (e.g., Argon Plasma Coagulation, clip placement) Stricture dilation Retrieval of retained video capsule
CE	Obscure Gastrointestinal Bleeding (OGIB) Suspected small-bowel Crohn's disease Screening for polyposis syndromes Evaluation of chronic abdominal pain/diarrhea	None
ERCP	Diagnostic role largely superseded by MRCP/EUS; reserved for select cases where non-invasive modalities are inconclusive. Cholestatic liver disease in neonates/infants (e.g., biliary atresia)	Common bile duct stone extraction Dilation and stenting of biliary/pancreatic strictures Management of biliary or pancreatic leaks Pancreatic duct drainage in chronic pancreatitis Drainage of pancreatic pseudocysts (often combined with EUS)
EUS	Evaluation of solid or cystic pancreatic lesions Unexplained biliary obstruction Subtyping of congenital esophageal stenosis Evaluation of subepithelial lesions, lymph nodes, or tumors	EUS-guided fine-needle aspiration/biopsy (FNA/FNB) Drainage of pancreatic pseudocysts Celiac plexus neurolysis (for pain management)

EGD, esophagogastroduodenoscopy; CE, capsule endoscopy; ERCP, endoscopic retrograde cholangiopancreatography; EUS, endoscopic ultrasound.

The safety of gastrointestinal endoscopy in children hinges on strict adherence to contraindications and a comprehensive understanding of potential adverse events (Table 5) (10, 101, 149). While diagnostic procedures such as gastroscopy, colonoscopy, and diagnostic EUS are generally safe with rare serious complications, the risk escalates significantly with the invasiveness and complexity of the intervention. High-risk procedures include therapeutic polypectomy via enteroscopy, post-ERCP pancreatitis, and EUS-guided drainage, whereas the foremost risk of capsule endoscopy is retention at an asymptomatic stricture. To maximize patient safety, all endoscopic procedures must be conducted by an experienced endoscopy in children team in a setting equipped with child-specific devices and under appropriate anesthesia and monitoring. Ultimately, rigorous pre-procedural evaluation (including imaging to exclude contraindications), refined technical skill, and diligent post-procedural observation are paramount to preventing and managing adverse events, thereby ensuring that the benefits of gastrointestinal endoscopy outweigh its risks for children.

**Table 5:** Contraindications and postprocedural adverse events of various endoscopic techniques in children.

Endoscopic technique	Primary contraindications	Potential adverse events
EGD	Absolute: To diagnose perforation (imaging is preferred), unstable airway or hemodynamics, suspected cervical spine injury. Relative: Severe coagulopathy, recent myocardial infarction, large esophageal diverticulum.	Common: Sore throat, hoarseness. Serious (Rare): Perforation, bleeding (especially post-intervention, e.g., variceal banding), sedation-related respiratory depression/hypotension, infection.
Colonoscopy	Absolute: Toxic megacolon, known or suspected bowel perforation, acute diverticulitis, hemodynamic instability. Relative: Recent bowel anastomosis (<7 days), uncorrected coagulopathy, poor bowel preparation.	Common: Abdominal bloating, transient hypotension. Serious: Perforation (most common during polypectomy or scope advancement), bleeding (post-polypectomy), sedation-related complications, infection, splenic or mesenteric tear (very rare).
Small-bowel endoscopy	Absolute: Hemodynamic instability, acute bowel perforation, peritonitis. Relative: Extensive intestinal adhesions, inability to tolerate prolonged anesthesia/sedation, untreated complete intestinal obstruction.	Perforation (especially after therapeutic polypectomy), pancreatitis, bleeding, intra-abdominal abscess, mucosal injury. Risk is higher in younger children (<10 years).
CE	Absolute: Known or suspected gastrointestinal obstruction, stenosis, or fistula (without prior patency assessment). Relative: Dysphagia (may require endoscopic deployment), cardiac pacemakers/defibrillators, pregnancy.	Primary Adverse Event: Capsule retention (most common at sites of unknown strictures, e.g., in Crohn's disease), often asymptomatic but may require surgical or endoscopic retrieval. Other: Aspiration (very rare, typically in patients with impaired swallowing).
ERCP	Absolute: Uncorrectable coagulopathy, acute non-biliary pancreatitis without evidence of obstruction. Relative: Unstable cardiopulmonary status preventing tolerance of the procedure, altered surgical anatomy (e.g., Billroth II gastrectomy, increasing difficulty and risk).	Most Common: Post-ERCP pancreatitis (PEP), particularly with pancreatic duct injection, sphincterotomy, and therapeutic procedures. Other: Hemorrhage (post-sphincterotomy), perforation, cholangitis, infection, sedation-related events.
EUS	Absolute: Esophageal stenosis preventing scope passage, known or suspected visceral perforation. Relative: Hemodynamic instability, coagulopathy (especially relevant for FNA).	Diagnostic EUS: Complication rate is very low, similar to standard EGD. EUS-FNA/FNB: Bleeding, infection (e.g., after cyst drainage), pancreatitis (after pancreatic puncture), perforation. Therapeutic EUS (e.g., drainage): Higher complication rate, including bleeding, perforation, stent migration/occlusion, and infection (including abscess formation).

EGD, esophagogastroduodenoscopy; CE, capsule endoscopy; ERCP, endoscopic retrograde cholangiopancreatography; EUS, endoscopic ultrasound; EUS-FNA, EUS-guided fine-needle aspiration; EUS-FNB, EUS-guided fine-needle biopsy.

## 7 Geographical inequalities in development

In the field of endoscopy in children, there remains a gap of several decades in overall capabilities between low- and middle-income countries (LMICs) and high-income nations, with development being highly uneven across regions. In Europe, under the coordinated leadership of the European Society for Paediatric Gastroenterology, Hepatology and Nutrition (ESPGHAN) and the European Society of Gastrointestinal Endoscopy (ESGE), a high-quality diagnosis and treatment system has been systematically established. This includes a competency-based, progressive training pathway supported by simulation training, e-learning platforms, and “train-the-trainer” programs. The region has also published the first comprehensive endoscopy in children guideline covering both diagnostic and therapeutic procedures (10, 150). These efforts have significantly enhanced the standardization, safety, and professionalism of endoscopy in children in Europe, providing an important model for global practice.

Asia has made remarkable progress in gastrointestinal endoscopy of children, particularly in the application of techniques and data accumulation. However, the absence of regional guidelines, unsystematic training, lack of unified quality control, and uneven distribution of resources remain key constraints. Future progress depends on regional collaboration, standardized training, guideline development, and optimized resource allocation to improve overall standards (151).

LMICs face substantial challenges in endoscopy in children, with development severely constrained by shortages of funding, equipment, specialized personnel, and infrastructure. There is a lack of systematic quality frameworks and underdeveloped data registry systems. Despite these obstacles, progress has been achieved in some regions through international organizational support, local pioneering efforts, and technological innovation, demonstrating commendable advances under difficult conditions (152).

## 8 Discussion

A critical challenge in gastrointestinal endoscopy in children lies in the lack of specialized equipment tailored to children's age, size, and anatomical characteristics. Despite strong recommendations for age/weight-appropriate endoscopic tools and children-specific monitoring devices, clinical practice often relies on adult-adapted equipment, compromising procedural safety and efficacy (67). Concurrently, the paucity of children-specific clinical trial data persists–most evidence is extrapolated from adult studies, leading to “very low” quality of evidence for key practices as highlighted by GRADE assessments (67, 68). Limited volumes of children with digestive diseases, even in tertiary centers, further restrict sample sizes, hindering robust validation of procedures and quality metrics (68, 84). Additionally, substantial inter-center heterogeneity is evident globally: training programs vary widely in duration, procedural thresholds, and assessment methods; clinical practices differ markedly in documentation completeness (e.g., bowel preparation quality), technical outcomes (e.g., ileal intubation rates), and adherence to quality standards (67, 68, 84).

High-quality endoscopy in children should embody the following characteristics: it must be safe and effective; it should focus on the experience and understanding of the child and their parents; it must adhere to standardized procedures based on consensus guidelines; and it should pursue continuous quality improvement through data feedback, peer comparison, and technological updates. In 2022, the North American Society for Pediatric Gastroenterology, Hepatology and Nutrition (NASPGHAN) and ESPGHAN jointly established the international Endoscopy in children Quality Improvement Network (PEnQuIN). This initiative aims to promote continuous quality improvement in endoscopy in children services worldwide by developing and implementing children-specific quality standards and metrics. Through a multi-level implementation framework involving endoscopists, endoscopic facilities (with standardized operating procedures, electronic reporting systems, and a culture of patient safety), and endoscopic procedures (including the establishment of multicenter collaborative networks and quality control registry systems), and leveraging technologies such as electronic medical records, artificial intelligence, and video recording, the automated collection, analysis, and feedback of quality indicators can be achieved (153). The PEnQuIN initiative represents a comprehensive, sustainable, and child-centered ecosystem for enhancing the quality of endoscopy in children globally, serving as a milestone in ushering the field into an era of high-quality practice.

Nevertheless, the implementation of high-quality endoscopy in children standards and indicators in clinical practice faces multiple challenges: difficulties in translating guidelines into practice; a widespread lack of automated data systems, hindering effective data collection and integration; limited application of emerging technologies such as AI and video assessment in endoscopy in children, coupled with a shortage of validated children-specific algorithms; and resistance among some endoscopists to performance feedback and quality improvement initiatives (154).

Therefore, we call for the establishment of a national endoscopy in children quality registry and collaborative network to facilitate multi-center data sharing and benchmarking; promote the adoption of digital and intelligent tools, such as AI-assisted diagnosis and evaluation systems; enhance endoscopist training and continuous education through modern teaching methods including video assessment and simulation training; and advocate for policy and financial support to integrate endoscopy in children quality improvement into hospital accreditation and health insurance evaluation systems. Guided by the PEnQuIN standards and adapted to local contexts, we should develop tailored endoscopy in children quality guidelines, working collectively to usher in a new era of evidence-based, child-centered, data-driven, and continuously improving endoscopy in children practice.
